# Neighboring Patch Density or Patch Size? Which Determines the Importance of Forest Patches in Maintaining Overall Landscape Connectivity in Kanas, Xinjiang, China

**DOI:** 10.3390/biology14070881

**Published:** 2025-07-18

**Authors:** Zhi Wang, Lei Han, Luyao Wang, Hui Shi, Yan Luo

**Affiliations:** 1 School of Business, Henan University of Science and Technology, Luoyang 471023, China; 9906212@haust.edu.cn (Z.W.); 9906174@haust.edu.cn (L.W.); 2 College of Animal Science and Technology, Henan University of Science and Technology, Luoyang 471023, China; 3 College of Tourism, Xinjiang University of Finance and Economics, No. 449 Beijing Road, Urumqi 830012, China; 4 College of Ecology and Environment, Xinjiang University, Urumqi 830017, China; luoyan505@xju.edu.cn

**Keywords:** biodiversity conservation, priority areas, patch configuration, stepping stone

## Abstract

The precise identification of priority areas for conservation based on connectivity can significantly enhance protection efficacy and mitigate biodiversity loss in fragmented landscapes. Priority area selection efforts are typically conducted in landscapes with a limited number of patches or simplified to focus on large patches, while landscapes with numerous patches are rarely explored. In this paper, we used a forest in Kanas, Xinjiang, China, as a case study to explore priority patches for conservation according to their contribution to maintaining overall landscape connectivity, as well as to assess how structural factors influence patch importance in connectivity, based on graph theory. We found that the rank of patches varied with patch importance indices (which can be used to calculate the contribution of individual patches to maintaining overall landscape). Dispersal distances were selected, as they placed different emphasis on the size and topological location of patches, and different types of links (binary or probabilistic connection) were used. One critical and seven important connected patches were identified as priority patches for conservation after taking multiple connectivity indices and dispersal distances into comprehensive consideration. In addition, neighboring patch density was the dominant factor that influenced patch importance for species with 50 and 100 m dispersal distances, while patch size contributed most for species with 200 m and longer dispersal distances; therefore, we suggested that neighboring patch density and patch size could be used to support efforts to identify priority patches. Overall, our results provide a unique perspective and a more simplified process for the selection of priority protected sites in patch-rich landscapes, allowing us to highlight which action is suitable for optimizing landscape connectivity and biodiversity conservation.

## Simple Summary

Traditional priority areas for conservation identification efforts could hardly deal with a landscape with numerous patches, or be simplified to focus on large patches. In Kanas, Xinjiang, China, we found the priority patch ranks varied with connectivity indices and dispersal distances used, and ultimately, one critical and seven important connected patches were identified as priority patches for conservation after considering multiple connectivity indices and dispersal distances. Besides, we found that neighboring patch density and patch size were the dominant factors that influenced patch importance for species with 50–100 m and ≥200 m dispersal distances, respectively. Therefore, neighboring patch density and patch size were recommended as proxies to identify priority patches, which could simplify the process of priority protected sites selection in patch-rich landscapes.

## 1. Introduction

Biodiversity loss has accelerated during the last 200 years [1], and nearly a third of Earth’s species would become extinct under the highest greenhouse gas emission scenario [2]; thus, halting biodiversity loss has become one of the major global challenges in the 21st century. Climate change, pollution, invasive species, overexploitation, and land use change have been proposed as the main threats to biodiversity, while land use change, which can cause habitat loss and degradation of species, contributes most to ongoing biodiversity loss [3]. Habitat loss and degradation not only directly reduce the population size of species but also decrease habitat connectivity and hinder many ecological processes, such as the search for new suitable habitat, dispersal of seeds and pollen, and gene flow between metapopulations [4,5,6]. Therefore, maintaining or increasing habitat connectivity has become recognized as a crucial process in the strategy for biodiversity conservation. It has been identified as such in government policy, such as the Aichi Target 15 of the post-2020 global biodiversity framework from the Convention on Biological Diversity [7] and the goal for 2050 related to the 2050 Vision for Biodiversity of the Kunming–Montreal Global Biodiversity Framework [8].

The majority of habitat loss is irreversible on a global scale, and species are compelled to retreat into numerous small and fragmented remnant habitat patches. Protecting all of the habitat patches or wildlife corridors is impossible and would lead to inefficient conservation efforts; therefore, conservation efforts frequently focus on well-connected patches as priority protection areas aim to improve connectivity [9]. Such a strategy has been demonstrated in many empirical studies; for example, enhancing habitat connectivity reduced the inbreeding of giant pandas (*Ailuropoda melanoleuca*) via gene flow and drove recovery [6], while the loss of the lake–river connection caused severe declines in molluscan biodiversity [10]. Traditionally, conservation efforts have usually been simplified to focus on large habitat patches over small ones [11], as it was believed that the former contributed more to maintaining overall landscape connectivity [12] and could support more species with large population sizes [13,14], while small habitat patches showed the opposite function [15]. However, in recent years, the disproportionately high value of small patches has been emphasized, as they can act as stepping stones to promote species’ dispersal between large but isolated patches. They can increase habitat availability for species [16,17] and serve as the last shelter for some endangered species [18].

More than 100 indices have been proposed to quantify overall landscape connectivity and/or the contribution of individual patches to maintaining it, in order to identify priority areas for conservation. These indices target different aspects of landscape connectivity and produce different spatial prioritization maps [9,15,19,20,21]. However, dispersal ability varies widely among taxa. For example, the mean dispersal distance is 50 m for most plants [22], while some mammals can easily move more than 1000 m across hostile matrices [23]; those different dispersal abilities would lead to a different contribution of individual patches to connectivity [9,24,25]. Although single indicators and representative species-based priority area selection can address the needs of multiple species, extending these results to the entire biota will neglect other crucial aspects of landscape connectivity and necessarily skew the outcome towards species with similar dispersal abilities. Therefore, in fragmented landscapes with numerous large and small patches, the selection of priority areas for conservation should be reconsidered to optimize the allocation of limited resources and maximize conservation outcomes.

With the proliferation of landscape connectivity studies, limitations with modeling tools based on graph-theoretic approaches have gradually emerged. These modeling tools can usually only process landscapes comprising hundreds or thousands of nodes (e.g., habitat patches or protected areas); a much higher number of nodes not only places very high requirements on computer processing capabilities but may also require several weeks to process the data. The millions of habitat patches would render large-scale landscape connectivity analysis expensive and impossible. Therefore, most studies were usually conducted on a small spatial scale with limited patches [12] or explored the connectivity between large patches [24], which may have led to some key small patches being excluded from analysis. Therefore, we should return to the characteristics of patches (such as patch size, shape, or topology) to explore the importance of patches in maintaining overall landscape connectivity.

Forests are crucial habitats for species and are ecosystems with the highest biodiversity on the earth’s terrestrial surface [26]. In this study, our main objectives were to rank the spatial conservation priorities of patches of a forest in Kanas, Xinjiang, China, based on a series of graph-based connectivity indices and multiple hypothetical species with different dispersal abilities. We also explored the role of patch characteristics in their contribution to maintaining overall landscape connectivity. Two questions were addressed: (1) Do the ranks of patch prioritization vary with connectivity indices and dispersal distances? (2) How do the structural factors of patches influence their importance in connectivity? Based on our findings, we provide further recommendations for improving the future identification of priority areas for conservation.

## 2. Materials and Methods

### 2.1. Study Area

**Figure 1:**
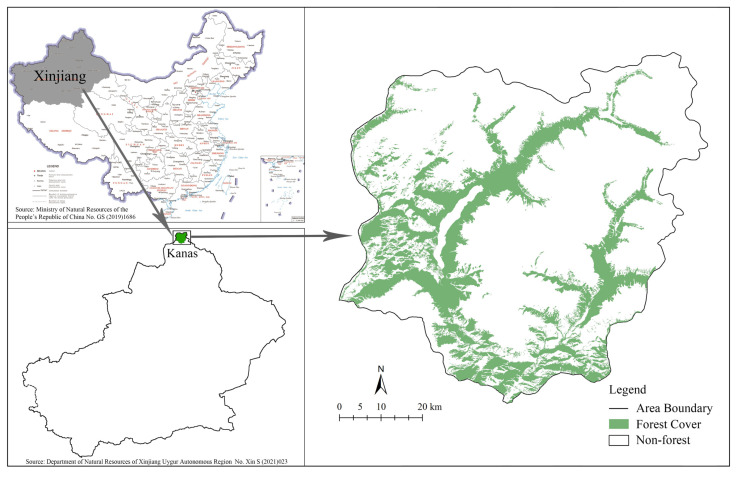
Location of study area and the distribution of forest patches in Kanas, Xinjiang Province, NW China.

Our study was carried out in Kanas, Xinjiang, northwest of China (86°41′–87°54′ E, 48°25′–49°29′ N). The region has an area of 5033.62 km^2^ and is at the border with Kazakhstan, Russia, and Mongolia (Figure 1). This region has a distinct temperate alpine mountain climate, with an average annual temperature, sunshine duration, and precipitation of −0.2 °C, 2157.4 h, and 1065 mm, respectively. The frost-free period is 80–108 d. It began to snow in October until April to May of the following year, with an average snow thickness of 0.5 m and a maximum depth of more than 1 m. The whole area is high in the north and low in the south, with an altitude of 1296–4374 m. It includes an alpine ice snow cold zone covered in modern glaciers and permanent snow at an altitude of above 3000 m, a periglacial subalpine cold tundra cushion meadow zone at an altitude of 2400–3000 m, a middle mountain cold temperate coniferous forest grassland zone at an altitude of 1300–2400 m, a hilly shrub meadow grassland zone at an altitude of 800–1500 m, and a piedmont alluvial proluvial plain oasis desert zone at an altitude of less than 800 m [27].

Kanas is located at the southern edge of the Altai–Sayan Ecoregion, which is one of the 200 priority ecoregions for global conservation [28], with a typical Taiga landscape and animal groups, and is the only national protected area (2201.62 km^2^) of China in the Palaeoearctic Euro-Siberian biogeographic region [29]. There are 39 species of mammals, 4 species of amphibians and reptiles, and 117 species of birds in this region. Among them, 27 species have been recognized in China’s key protected species lists, including snow leopard (*Panthera uncia*), wolverine (*Gulo gulo*), Eurasian Lynx (*Lynx lynx*), red deer (*Cervus canadensis*), Moose (*Alces alces*), and Eurasian Otter (*Lutra lutra*). In addition, Kanas is the only distribution area in China of Altai Brown Frog (*Rana altaica*), Rock Ptarmigan (*Lagopus muta*), and Western Capercaillie (*Tetrao urogallus*) [29]. There are 1491 species of spermatophyte distributed in Kanas, including ubiquitous species such as *Larix sibirica*, *Populus tremula*, *Betula pendula*, and *Picea sibirica*, and endemic species such as *Rives saxatile*, *L.cearulea var. altaica*, *Vaccinium vitsidaea*, *Betula rotundifolia*, and *Betula humilis* [30].

### 2.2. Data Sources and Processing

The Landsat enhanced thematic mapper remote sensing image of Kanas in 2022, with a resolution of 30 m/pixel, was downloaded from the United States Geological Survey website (https://glovis.usgs.gov). Then, the supervised classification tool in ENVI 5.3 (https://envi.geoscene.cn/) software was used to process the above remote sensing image to obtain the land cover (forest, high-coverage grassland, medium-coverage grassland, low-coverage grassland, water, construction land, glacier and perennial snowfield, and unused land) of Kanas. As only forest and non-forest areas were needed, 5034 points were randomly generated within the study area to assess the accuracy of the forest map by comparing it with Google Earth Map. It was concluded that the overall accuracy was 95.13%. The area of each forest patch and the distances between all forest patches were calculated using the Patch analyst extension of ArcGIS 10.5 (https://www.esri.com/).

A series of graph-theoretic connectivity measures were used to assess the overall forest landscape connectivity in Kanas, including the Probability of Connectivity (*PC*), the Integral Index of Connectivity (*IIC*), the Landscape Coincidence Probability (*LCP*), and the Number of Components (*NC*) (a component is a set of patches in which a path exists between patches) [31,32]. These indices have been most extensively used in landscape connectivity studies [21] and focus on different aspects of connectivity: *PC* evaluates connectivity by assessing the probability of movement or dispersal of species, considering both direct and indirect potential paths; *IIC* highlights habitat availability, and only direct paths between patches are used; *LCP* pays attention to the reachability between components, without information on the internal connectivity of components; *NC* focuses on the topological position of patches and ignores their sizes. For individual patches, their contributions to maintaining overall landscape or patch importance were calculated based on changes in the above four overall landscape connectivity measures when a given patch was hypothetically removed. These were marked as *dPC*, *dIIC*, *dLCP*, and *dNC*, respectively [32]. The overall landscape connectivity increased with *PC*, *IIC*, and *LCP*, but decreased with *NC*. Consistently, a patch with larger values of *dPC*, *dIIC*, and *dLCP* or a smaller value of *dNC* would have higher patch importance.

The perceived ability varies with species in the same landscape structure, and the landscape connectivity depends on the specific dispersal distances of each species [9]. Therefore, by following the research conducted at the Bogda World Natural Heritage Site in Xinjiang [25] and considering the species living in Kanas, seven hypothetical median dispersal distances were used in this study: 50, 100, 200, 500, 1000, 2000, and 5000 m. These distances covered most species’ dispersal distances, such as those of plants and the Brown frog (50 m and 500 m, respectively) [22,33], as well as some more mobile species, such as Moose and red deer, the dispersal distances of which may be several kilometers [23]. All of the overall landscape connectivity indices (*PC*, *IIC*, *LCP,* and *NC*) and patch importances indices (*dPC*, *dIIC*, *dLCP*, and *dNC*) were calculated for these selected dispersal distances.

To explore the difference in priority patches for conservation derived from the value of different patch importance indices, we not only calculated Spearman’s rank correlations for the patch importance values of all patches for each pair of indices but also compared the inconsistency in the rankings of the top 10 patches with the highest importance. The conservation priority map, showing patch importance in maintaining overall connectivity, was calculated using k-means clustering analysis, considering all of the patch importance indices.

Redundancy analysis (RDA) was used to explore the relationship between patch structural factors and patch importance, as well as how patch structural factors affect patch importance. The patch structural factors used in this study were patch size (PS), shape index (SI), mean inter-patch distance (MID), and neighboring patch density (NPD), with the latter two factors representing the patch configuration. For a given patch, the value of PS was equal to the area of this patch; the value of SI was calculated as the ratio of its perimeter to the minimum possible perimeter (circular reference) for a patch of the same area; the value of MID was determined by the average Euclidean distance between this patch and all other patches; the value of NPD was the number of patches within our selected dispersal distances. Before redundancy analysis, a correlation analysis was performed with the four patch structural factors to exclude multicollinear variables, as all structural factors had a VIF < 10 (ranged from 1.01 to 7.71). The results indicated that these four factors were uncorrelated, and thus, all of them were used in the redundancy analysis. Forward selection was used to calculate each structural factor’s contribution in RDA. The overall landscape connectivity and patch importance were measured by Conefor Sensorode 2.2 [34], RDA was conducted with CANOCO 4.5 software [35], and Spearman’s rank correlation analysis and k-means clustering analysis were performed in SPSS 25.0.

## 3. Results

### 3.1. Quantifying Overall Landscape Connectivity of Forest Patches

The land cover classification of Kanas shows that the forest landscape is composed of 2884 patches, ranging from 0.05 ha to 58,842.21 ha, with a total area of 122,612.12 ha. The overall landscape connectivity of the forest in Kanas increased with the seven selected dispersal distances, as the values of *PC*, *IIC*, and *LCP* increased with dispersal distance, while the value of *NC* decreased (Table 1). In particular, the value of *NC* reached 1 at a dispersal distance of 5000 m (Table 1), which means that species with a dispersal ability of 5000 m could reach any other forest patch in our study area from an initial patch.

### 3.2. Comparing Patch Importance Based on Different Landscape Connectivity Indices and Identifying Important Patches

Each pair of patch importance values measured by *dPC*, *dIIC*, and *dLCP* was strongly (0.6 ≤ r < 0.8, *p* < 0.01) or very strongly (r ≥ 0.8, *p* < 0.01) correlated, and the correlation coefficients among them showed an increased tendency with dispersal distances (Table 2), indicating that the patch prioritization identified based on these three indices were quite consistent, especially for species with higher dispersal ability. However, patch prioritization measured by *dNC* was significantly different from the former three indices, and their correlation gradually decreased with dispersal distances; only two strong correlation coefficients (0.6 ≤ |r| < 0.8, *p* < 0.01) were found between *dNC* with *dLCP* and between *dNC* and *dIIC* for species with a dispersal distance of 50 m (Table 2). At 50 and 100 m dispersal distances, the correlations between *dPC* and *dIIC*/*dLCP* were weaker than those between *dIIC* and *dLCP*, and the *dPC*-*dNC* correlations were also lower than both *dIIC*-*dNC* and *dLCP*-*dNC* (Table 2).

**Figure 2:**
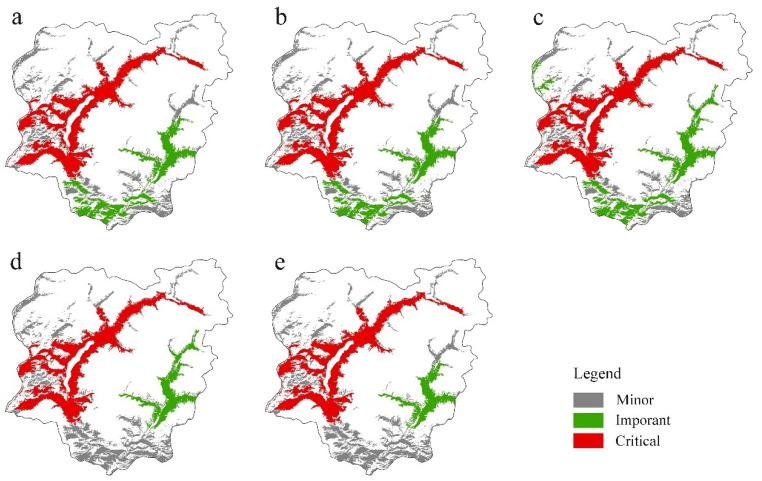
Importance of the forest patches in Kanas based on *dPC*, *dIIC*, *dLCP*, and *dNC* by k-means clustering analysis, for dispersal distances of 50 m and 100 m (**a**), 200 m (**b**), 500 m (**c**), 1000 m (**d**), and 2000 m and 5000 m (**e**).

The ranking of the top 10 most important forest patches showed high inconsistency between both selected indices and dispersal distances; the values between indices showed the opposite trends for the same patch as dispersal distances increased (Table S1). For example, patch # 2881 ranked second at a dispersal distance of 50 m but dropped to fourth or fifth as the dispersal distance increased, while the rank of patch # 1196 increased from fifth to second. *dNC* only identified seven and two important patches at dispersal distances of 2000 m and 5000 m, respectively (Table S1), suggesting that using single indices to identify priority areas could not satisfy the requirement of biodiversity conservation for multiple species and objectives. All of the four indices suggested that patch # 2499 had the highest importance in maintaining overall forest landscape connectivity at all selected dispersal distances (Table S1), because it had the largest size and adjoined the highest number of other patches; thus, it should be set as the highest-level protected patch. In addition, according to the result of patch clustering analysis, patch # 2499 in the Khanas Nature Reserve was classed into the first group across all dispersal distances, and five, five, four, six, two, one, and one patches were classed into the second group for the seven selected dispersal distances, respectively, while the remaining patches were classed into the third group (Figure 2). Considering their values of patch importance, patch # 2499 should be marked as a critical one for conservation, and patches belonging to the second group should be recognized as important patches for priority protection (Figure 2).

### 3.3. The Explanation Power of Each Structural Factor on Patch Importance

The forward selection results of RDA showed that the total adjusted explained variance of forest patch importance in Kanas explained by structural factors varied with dispersal distances and ranged from 87.0% to 97.3% (Table 3). NPD contributed most to the total explained variance for species with dispersal distances of 50 m (81.5%) and 100 m (84.6%), followed by PS (5.4% and 3.8%, respectively), while both SI and MID only contributed 0.1% or lower (Table 2). For species with dispersal distances of 200 to 500 m, PS contributed most to the total explained variance (≥94.6%), followed by NPD (ranging from <0.1% to 1.1%) and SI (ranging from <0.1% to 0.4%), and the contribution of MID was <0.1% (Table 2).

**Figure 3:**
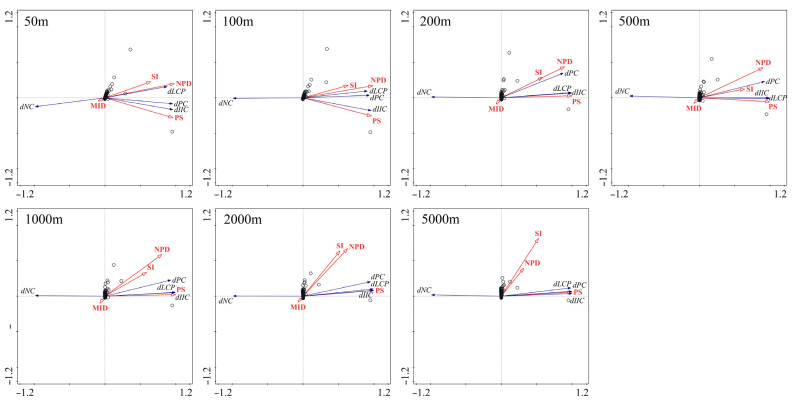
RDA biplot of patch structural factors (PS, NPD, SI, and MID) and patch importance indices (*dPC*, *dIIC*, *dLCP*, and *dNC*) for the seven selected dispersal distances.

**Figure 4:**
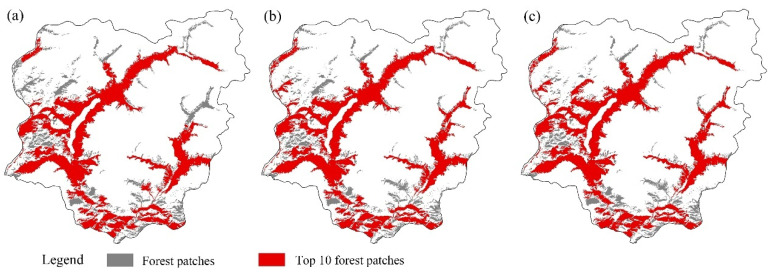
Top 10 patches with the highest value of NPD ((**a**) for 50 m and (**b**) for 100 m dispersal distances) and PS (**c**).

In general, MID was negligible in relation to the four landscape connectivity indices, while SI, NPD, and PS were positively related to *dLCP*, *dIIC*, and *dPC* and negatively related to *dNC* (Figure 3), indicating that patch importance increased with SI, NPD, and PS. As NPD contributed most to maintaining overall landscape connectivity at short dispersal distances (50 m and 100 m), and PS contributed most at long dispersal distances (≥200 m), the top 10 patches with the largest NPD (50 m and 100 m dispersal distance, respectively) and PS were shown in Figure 4. A total of 12 patches were identified, with 8 patches appearing in both the top 10 PS and NPD maps simultaneously. These patches should receive more attention than the other four patches when formulating a protection plan.

## 4. Discussion

### 4.1. Spatial Mismatch Among Priority Patches for Different Connectivity Indices and Dispersal Distances

Identifying priority areas with high contributions to maintaining overall landscape connectivity would optimize biodiversity conservation efficiency [25]. Therefore, it is crucial to have a comprehensive knowledge of landscape connectivity before conservation decision-making processes. Similarly to the study conducted in three reserve networks in Finland [36] and a goshawk (*Accipiter gentilis*) habitat in Spain [37], we also found an obvious difference in patch priority conservation order among the results derived from the different patch importance indices. This spatial mismatch phenomenon could be explained by the fact that those indices place a different emphasis on patch size and topological location of patches (i.e., links), as well as the model to quantify links (binary vs. probabilistic connection model): *dNC* was calculated based only on the links between patches (binary connection model), while *dPC*, *dIIC*, and *dLCP* consider the patch size and links (probabilistic, binary, and binary, respectively) together, but *dPC* and *dIIC* consider the links at the patch level, and *dLCP* further considers the links at both the patch and component levels [32]. As patch size is an important factor influencing patch importance, it is reasonable that correlations of the values among *dPC*, *dIIC*, and *dLCP* were much stronger than with that derived from *dNC* in our study. On the other hand, the correlation coefficients of the values between *dNC* and *dPC*, *dIIC*, and *dLCP* were stronger at dispersal distances of 50 and 100 m compared to 200 m and greater. This may be because patch importance derived from the latter three indices was influenced by both links and patch size at shorter dispersal distances, but mainly by patch size at longer dispersal distances. In addition, at dispersal distances of 50 and 100 m, *dPC* correlated less strongly with *dIIC*/*dLCP* and *dNC* compared to both *dIIC*-*dLCP* and *dIIC*/*dLCP*-*dNC* relationships. This result possibly indicates that the use of a probabilistic connection model made *dPC* more sensitive to patch size, while the other three indices, which used a binary connection model, were more sensitive to links.

Indeed, *PC* and *IIC*, which have given rise to *dPC* and *dIIC*, have been recommended as the best indices for integrating connectivity in landscape conservation planning as they combine graph theory and habitat availability metrics [38]. However, they stress the significance of large patches, as they have a size-weighted distance component, and ignore small patches, which play a critical role as stepping stones. On the other hand, *dNC* is biased in topological aspects, which would inevitably lead to an overestimation of the role of small patches if it was used alone to quantify patch importance; and our results showed that *dNC* could only identify seven and two important patches at dispersal distances of 2000 m and 5000 m, respectively, indicating that the ability of *dNC* to identify important patches is limited for species with long dispersal distances. Therefore, we suggest combining the outcomes of multiple connectivity indices, taking the patch size and topological location into account, which would give a better solution than relying on a single index when identifying priority patches. *dPC* was suitable for tracking the quantities of species’ movement [32], *dIIC* revealed the process of gene flow [39], *dNC* gave a better basis for identifying critical small patches [15], and *dLCP* provided a view of connectivity at the component level [36].

Apart from the connectivity indices, we also found that the influence of the dispersal ability of the selected focal species on the importance of a given patch was variable: the values increased at certain dispersal distances but decreased at others, leading to significant changes in patch importance rankings. A similar phenomenon has also been reported in many other studies, such as at the Bogda World Natural Heritage Site [25], and a rural landscape in the tropical lowlands of Mexico [24]. This inconsistency may be due to differences in species’ dispersal abilities, which would change the relative spatial positions between different patches, which determines whether they are connected or not. In fact, it is impossible to obtain all ecological data of a given ecosystem, and unforeseen events are also inevitable. Thus, an effective conservation network should consider as many representative connectivity indices and species as possible. In doing so, we concluded that patch # 2499 was the most critical patch in maintaining overall landscape connectivity, as it had the highest value of patch importance across all selected indices and hypothetical species. Thus, it should receive the highest attention during conservation efforts. We also identified seven additional patches, marked as important patches, which should also be included in the list of priority patches for conservation.

### 4.2. The Influence of Structural Factors on Patch Importance

More in-depth knowledge regarding the role of patch structural factors in determining patch importance would improve the effectiveness of fragmented landscape protection. We found that patch importance was positively related to PS, NPD, and SI, indicating that a patch with a larger area, higher edge-to-area ratio, or more neighboring patches would contribute more to maintaining overall landscape connectivity. This result was expected, as larger patches not only provided more habitats for species but also transmitted more ecological fluxes [35]; a higher edge-to-area ratio may make patches easier to find [40]; more neighboring patches means lower patch isolation and therefore higher habitat availability [41]. MID was used to measure the location of patches in the landscape. We surprisingly found that MID was negligible in relation to patch importance. A possible explanation for this might be that MID was measured as the average distance to all other patches, meaning that patches with lower MID values are more likely to be centrally located within the landscape, instead of simply having shorter distances to nearby patches. Species dispersal is more likely to take place along the shortest path across hostile matrices, so patches with low MID values do not necessarily act as stepping stones or attract more species.

Patch configuration and patch size have been proposed to be the most prominent factors in landscape connectivity, but their relative importance varied with dispersal distances [42]. Short-distance dispersers were mostly restricted to isolated patches, while long-distance dispersers reached target patches mainly through their dispersal ability, rather than relying on other patches as stepping stones; therefore, patch size was more important for short- and long-distance dispersers. However, for intermediate-distance dispersers, whose dispersal ability is near the percolation threshold, path configuration played the most important role, as the presence of other patches would improve the long-distance dispersal behavior of species and increase their habitat availability. Laita et al. [36] also found, in a herb-rich forest and spruce mire networks, that the contribution of PS was lower at 1 and 2.5 km compared to at shorter or longer dispersal distances, and they suggested the importance of patch configuration at this scale. However, our results showed that NPD (representing patch configuration) was the dominant factor for species with dispersal distances of 50 and 100 m, whereas PS was the dominant factor for dispersal distances of 200 m or more. The relative importance of patch configuration over size emerged in shorter distances in our study. This may be because the patch density (number of patches per landscape area) and habitat proportion in our study area were higher than those of Laita et al. [36], i.e., 0.57 vs. 0.08 and 0.31; and 24.35% vs. 0.16% and 0.46%. The higher patch density and habitat proportion in our study area mean shorter distances between nearby patches and greater habitat availability for short-distance dispersers, thus lowering the threshold value for percolation transition. On the other hand, at 50 and 100 m dispersal distances, there were 1359 and 648 components in our study area, respectively, and most of these components were composed of a couple of patches. This indicated that the patches in our study area were neither isolated completely from one another nor sufficiently connected to form one or several components at this scale, so it is reasonable that NPD had the greatest influence on patch importance.

Qi et al. [12] found that SI was the least important factor for patch importance in their study involving four sites. Our results also demonstrated that SI contributed a little to that, a finding which can be attributed to the low level of correlation between the SI values and patch importance indices in both studies. In addition, the key requirement for species diffusion is effective connection, which is related to the distance or corridor between patches. Although a patch with a more complex shape can increase encounter rates [17], it does not directly reflect the spatial relationship between patches. For example, if a patch is beyond the reach of individuals from other patches, its role in overall landscape connectivity would be stable irrespective of its shape. Furthermore, as mentioned above, providing intra-habitat functions and transmitting ecological fluxes are critical components of a patch’s role in connectivity, both of which are determined by patch size [38]; however, patches with high SI values did not always have a large size. In this situation, it is unsurprising to find that the contribution of SI to patch importance was much lower compared to NPD and PS.

### 4.3. Suggestions for Management

In order to optimize the limited resource allocation and increase the efficiency of area-based conservation efforts, it is of key importance to identify which key patches play a crucial role in landscape connectivity. Therefore, we suggest that multiple patch importance indices and dispersal distances should be used together to identify priority conserved patches. One critical and seven important patches in Kanas (Figure 2) were identified using this method, indicating the patches to which most resources should be devoted. However, identifying priority patches using frequently used indices is usually complex or only suitable for small-spatial-scale management. Considering the trade-offs between simplicity and ecological realism, using these structural factors that have the highest influence in patch importance as proxies, such as NPD and PS in this study, would be both effective and practical, especially in large-scale landscapes or data-limited settings. In this study, we found that NPD and PS were dominant factors in patch importance, for species with dispersal distances of 50 to 100 m and ≥200 m, respectively. Thus, patches with top 10 values of NPD and PS, as shown in Figure 4, could be used to support efforts to identify priority patches. Furthermore, restoring as many habitats as possible is likely the best strategy for fragmented landscapes [43], but the location for restoration will depend on the dispersal distances of focal species. For short-distance dispersers, which are usually confined to isolated patches, it is recommended to prioritize restoration near contiguous patches to connect them into larger patches or to increase the size of isolated patches. For intermediate-distance dispersers, optimizing patch configuration, such as by establishing new small patches as stepping stones or building ecological corridors between components, which are easier to implement and have lower costs [9], would increase landscape connectivity and the dispersal range of species. For long-distance dispersers, increasing the size of large patches or creating new large patches should be more effective.

In this study, we found that the relative importance of patch configuration over size in patch importance was influenced by the threshold value of percolation transition, but its mechanism was unclear. Thus, we should explore the relationship between patch configuration, patch size, patch density, habitat proportion, and other landscape properties in future work. In addition, we did not consider the difference in quality between patches apart from their size; however, flora characteristics such as the types of forest and density of trees per patch directly influence species richness and population density, as well as the ecological flow; therefore, considering these factors in patch importance quantification and priority patch identification efforts would better align with actual ecological processes and help promote biodiversity conservation.

## 5. Conclusions

Identifying priority patches for conservation is a useful strategy for buffering biodiversity loss in fragmented landscapes. In this study, we identified one critical and seven important patches for conservation based on their contribution to maintaining overall landscape connectivity in the forest of Kanas, Xinjiang, China, and highlighted the importance of using multiple connectivity indices and dispersal distances. In addition, we demonstrated NPD as the dominant factor that influences patch importance for species with dispersal distances of 50 and 100 m, while PS was the dominant factor for dispersal distances of 200 m and longer; therefore, NPD and PS were recommended as proxies to identify priority patches. Finally, based on the contribution of NPD and PS in patch importance, we suggested a triple strategy of connecting contiguous patches or increasing the size of isolated patches for short-distance dispersers, establishing new stepping stones or corridors for intermediate-distance dispersers, and increasing the size of large patches or creating new large patches for long-distance dispersers. Future biodiversity conservation work should consider the relative importance of patch size and patch configuration in relation to the dispersal ability of focal species.

## Figures and Tables

**Table 1:** Overall landscape connectivity of forest patches in Kanas.

Dispersal Distance	50 m	100 m	200 m	500 m	1000 m	2000 m	5000 m
*PC* (%)	2.71	3.47	4.19	4.95	5.35	5.61	5.79
*IIC* (%)	2.23	2.29	2.40	2.57	2.65	2.73	2.84
*LCP* (%)	4.59	4.87	5.62	5.81	5.92	5.93	5.93
*NC*	1359	648	218	38	9	2	1

**Table 2:** The correlation coefficients of patch importance pairwise comparisons based on different landscape connectivity indices.

Dispersal Distance	50 m	100 m	200 m	500 m	1000 m	2000 m	5000 m
*dNC* vs. *dLCP*	−0.644 **	−0.522 **	−0.192 **	−0.197 **	−0.192 **	−0.083 **	−0.046 *
*dNC* vs. *dIIC*	−0.615 **	−0.504 **	−0.19 **	−0.188 **	−0.190 **	−0.082 **	−0.046 *
*dNC* vs. *dPC*	−0.331 **	−0.296 **	−0.133 **	−0.187 **	−0.157 **	−0.082 **	−0.046 *
*dLPC* vs. *dIIC*	0.986 **	0.992 **	0.977 **	0.979 **	0.977 **	0.985 **	0.991 **
*dLCP* vs. *dPC*	0.668 **	0.762 **	0.784 **	0.912 **	0.946 **	0.980 **	0.993 **
*dIIC* vs. *dPC*	0.691 **	0.784 **	0.845 **	0.924 **	0.955 **	0.975 **	0.986 **

Note: ** mean *p* < 0.01, * mean *p* < 0.05.

**Table 3:** Percentage of forest patch importance explained by structural factors.

Dispersal Distances	50 m	100 m	200 m	500 m	1000 m	2000 m	5000 m
PS	5.4	3.8	96.1	94.6	96.4	96.6	96.9
NPD	81.5	84.6	0.4	1.1	0.2	0.4	<0.1
SI	<0.1	0.1	0.1	0.4	<0.1	0.2	0.4
MID	<0.1	-	<0.1	<0.1	<0.1	<0.1	-
Total adjusted explained variance	87.0	88.6	96.6	96.1	96.7	97.3	97.3

Note: - means MID showed collinearity with other factors and was thus excluded from analysis.

## Data Availability

The data presented in this study are available on request from the author; the data are also part of an ongoing study.

## Supplementary Materials

The following supporting information can be downloaded at: https://www.mdpi.com/article/10.3390/biology14070881/s1, Table S1: The top 10 most important patches ranked according to the 4 indices studied, N means the number of patches.

## Abbreviations

The following abbreviations are used in this manuscript:

PS	Patch size
SI	Shape index
MID	Mean inter-patch distance
NPD	Neighboring patch density
