# Supplementary material for: Neighboring Patch Density or Patch Size? Which Determines the Importance of Forest Patches in Maintaining Overall Landscape Connectivity in Kanas, Xinjiang, China
Source: Biology (Basel). 2025 Jul 18;14(7):881. doi: 10.3390/biology14070881 (PMC12292692; doi:10.3390/biology14070881)
Supplement: Supplementary file 1 [file biology-14-00881-s001.zip › biology-3709986-supplementary.pdf]

Table S1 The top 10 most important patches ranked according to the 4 indices studied, N means the number of patches.

Rank	50m								100m							
	N	*dPC*	N	*dIIC*	N	*dLCP*	N	*dNC*	N	*dPC*	N	*dIIC*	N	*dLCP*	N	*dNC*
1	2499	85.22	2499	86.94	2499	84.12	2499	-11.77	2499	84.19	2499	86.67	2499	83.71	2499	-29.78
2	2881	27.65	2881	20.99	2881	50.79	487	-6.11	487	36.91	487	19.97	487	50.11	487	-10.96
3	487	26.29	487	19.76	487	50.21	1196	-3.83	2881	32.92	1196	14.26	2879	35.92	1196	-9.88
4	2879	14.68	1196	13.83	2879	35.34	2881	-2.13	2879	23.29	2881	13.27	1196	31.24	2335	-3.86
5	1196	14.14	2879	12.29	1196	30.39	2878	-1.47	1196	20.76	2879	12.65	769	30.86	2878	-3.4
6	769	10.59	769	9.95	769	30.23	2879	-1.4	769	16.75	769	10.29	2878	6.73	2879	-3.4
7	2878	4.69	2878	2.86	2878	6.59	2335	-1.32	2878	6.07	2878	2.94	2881	6.65	2881	-3.24
8	569	3.37	2133	2.14	2063	5.01	2563	-1.32	2063	3.32	2063	2.21	2063	5.43	2563	-3.09
9	1702	2.52	2063	2.06	2133	2.89	2133	-0.81	569	2.62	2133	2.12	2133	2.82	2063	-2.62
10	2063	2.19	1702	1.49	2873	2.85	630	-0.66	2133	2.46	1702	1.45	2873	2.81	2133	-1.85
**Rank**	**200m**								**500m**							
	N	*dPC*	N	*dIIC*	N	*dLCP*	N	*dNC*	N	*dPC*	N	*dIIC*	N	*dLCP*	N	*dNC*
1	2499	83.92	2499	86.57	2499	84.37	2499	-82.11	2499	84.12	2499	86.72	2499	84.7	2499	-276.32
2	487	43.2	487	20.61	487	49.52	1196	-24.77	487	45.77	487	18.78	1196	28.75	1196	-76.32
3	2881	28.63	1196	14.25	2879	34.25	487	-21.1	1196	27.64	1196	14.61	487	13.85	2335	-52.63
4	2879	28.39	2879	12.86	1196	29.44	2335	-14.22	2879	26.93	2881	13.56	2335	7.29	487	-42.11
5	1196	25.11	2881	12.57	2335	7.26	2878	-9.17	2881	18.23	2879	9.77	2109	7.26	2063	-34.21
6	769	16.4	769	3.25	2109	7.23	2563	-7.8	769	10.18	2335	2.96	2881	6.09	2563	-21.05
7	2878	6.61	2878	2.8	2122	7.18	2879	-7.8	2878	6.31	2109	2.87	2878	5.17	2879	-21.05
8	2063	4.16	2063	2.23	2881	6.23	2881	-7.8	2063	4.7	2878	2.77	2063	5.07	2881	-21.05
9	2133	2.72	2335	2.21	2878	5.31	2063	-6.88	2335	4.28	2133	2.3	2879	4.13	2133	-18.42
10	2873	2.22	2109	2.1	2063	5.09	2873	-5.96	2133	2.96	2063	2.26	2133	3.18	2870	-13.16
**Rank**	**1000m**								**2000m**							
	N	*dPC*	N	*dIIC*	N	*dLCP*	N	*dNC*	N	*dPC*	N	*dIIC*	N	*dLCP*	N	*dNC*
1	2499	84.34	2499	86.4	2499	84.79	2499	-577.78	2499	84.41	2499	86.39	2499	84.59	2499	-1200

2	487	41.66	487	17.46	1196	28.58	1196	-177.78	487	33.47	487	15	1196	27.74	1196	-350
3	1196	28.19	1196	14.43	487	13.21	2063	-100	1196	27.73	1196	14.1	487	13.2	2063	-50
4	2879	20.68	2881	11.9	2335	7.23	487	-44.44	2879	14.26	2881	7.13	2878	5.11	2213	-50
5	2881	12.2	2879	6.4	2878	5.12	2335	-33.33	2881	8.48	2879	4.22	2063	4.36	2563	-50
6	769	5.98	2335	3.68	2063	4.99	2563	-33.33	2335	6.35	2335	4	2881	4.2	2687	-50
7	2878	5.87	2878	3.24	2881	4.2	2133	-22.22	2878	5.54	2878	3.13	2563	4.01	2740	-50
8	2335	5.75	2133	2.35	2879	4.05	2363	-22.22	2063	4.75	2133	2.11	2879	4	-	-
9	2063	4.82	2063	2.22	2563	4.01	2635	-22.22	769	3.3	2063	2.02	2335	3.16	-	-
10	2133	3.06	2563	1.61	2133	3.38	2756	-22.22	2563	3.15	2563	2	2133	2.52	-	-

**Rank**	**5000m**							
	**N**	***dPC***	**N**	***dIIC***	**N**	***dLCP***	**N**	***dNC***
1	2499	83.45	2499	85.15	2499	83.69	2499	-300
2	1196	25.94	1196	14.74	1196	22.48	1196	-100
3	487	23.5	487	12.32	487	13.19	-	-
4	2879	8.68	2881	3.66	2878	5.1	-	-
5	2881	5.98	2878	3.18	2881	4.2	-	-
6	2335	5.7	2335	2.88	2063	4.18	-	-
7	2878	5.29	2879	2.75	2879	4	-	-
8	2063	4.53	2063	2.07	2335	3.16	-	-
9	2563	3.28	2133	1.93	2563	2.57	-	-
10	2133	2.87	2563	1.54	2133	2.52	-	-

Note: - mean there are no more patches could be identified as priority conserve patches as their value of dNC was 0.
